# On the explicit use of experimental images in high resolution cryo-EM reﬁnement

**DOI:** 10.12688/f1000research.19230.2

**Published:** 2019-11-28

**Authors:** Jacqueline Cherfils, Jorge Navaza

**Affiliations:** 1 Laboratoire de Biologie et Pharmacologie Appliquée, CNRS and Ecole Normale Supérieure Paris-Saclay, Cachan, 94235, France

**Keywords:** cryo-EM, X-ray crystallography, computational method, model refinement, structural biology

## Abstract

Single particle cryogenic electron microscopy (cryo-EM) is transforming structural biology by enabling the analysis of difficult macromolecular specimens, such as membrane proteins or large complexes with flexible elements, at near atomic resolution with an accuracy close to that of X-ray crystallography. As the technique continues to improve, it is important to assess and exploit its full potential to produce the most possible reliable atomic models. Here we propose to use the experimental images as the data for refinement and validation, instead of the reconstructed maps as currently used. This procedure, which is in spirit quite similar to that used in X-ray crystallography where the data include experimental phases, should contribute to improve the quality of the cryo-EM atomic models.

## Discussion

In this short communication the word model refers to the macromolecular structure represented by its atomic coordinates, and the word refinement refers to the process of improving the model so that the calculated data based on the model best fit the observed data.

Single-particle cryo-EM has recently joined the circle of techniques that allow macromolecular structure determination at almost atomic resolution. The technique presents a clear advantage over X-ray crystallography in that it allows the structural analysis of particles that are difficult or impossible to grow into crystals, such as very large complexes, membrane proteins or proteins with flexibles portions, and is therefore gaining a prevalent role in the determination of biological macromolecular structures ^1^. As a relatively young method, computational methods to interpret images and derive atomic models continue to be developed and optimized ^2, 3^. In that regard, a recent comparison of X-ray and cryo-EM maps calculated at the same resolution, together with the corresponding atomic models, showed that although the appearance of the maps was quite comparable between the two techniques, X-ray crystallography maps were more detailed and the atomic models fitted into them were more accurate ^4^. To make the comparison on a fair basis the X-ray electron densities were calculated with experimental phases (SAD, heavy-atom) and improved by density modification. In this way the maps produced by both techniques were model-free. The accuracy and level of detail of the maps were assessed by fitting the already determined atomic structures into them. These results begged for microscopists to continue to improve the accuracy and performance of methods for map improvement and model refinement, in order to produce atomic models that meet the same quality standards as in X-ray crystallography.

The cryo-EM and X-ray crystallography experimental techniques are very different but the final stages of the structure determination process are similar. An important difference between crystallography and cryo-EM as techniques to reconstruct scattering densities is that in cryo-EM it is possible, in principle, to obtain a reconstruction starting from the raw experimental data without imposing any model, under the assumption that the data are projections of similar particles. If we look at the cryo-EM data in reciprocal space the similarities and differences with X-ray data become apparent. The actual experimental data in cryo-EM are two-dimensional images related to the projections of the particle electrostatic potential, along different directions. According to the projection-slice theorem, the Fourier transform of the two-dimensional experimental image corresponds to a central section of the three-dimensional particle Fourier transform multiplied by the contrast transfer function (CTF), a well-defined mathematical function that introduces a modulation in reciprocal space and restricts the data to annular domains delimited by the zero-crossings of the function. So, while in X-ray crystallography the diffraction experiment gives amplitudes of Fourier coefficients for points in a known reciprocal lattice, in cryo-EM the images give, after CTF correction, complex Fourier coefficients in central planes whose relative orientations in reciprocal space are unknown. The accurate determination of the CTF and the assignment of orientations and centers to the images is the task of the reconstruction procedure which, in principle, does not depend on any model or template. In this regard, the cryo-EM map is somehow equivalent to an experimentally phased X-ray crystallography map in the sense that the required orientations and phases are determined from the sole experimental data. With near atomic resolution data, these maps allow the construction of initial atomic models. In X-ray crystallography, model phases are used to update the map during model building and refinement, keeping the experimental structure factors—the data—unchanged. Thereby, the crystallographic model is improved as the map becomes clearer in both the particle and solvent regions based on feedback from calculated phases. In cryo-EM, the reconstruction is considered as experimental data and kept unchanged during model building and refinement ^5^. In keeping with elementary ideas of data, it seems natural that the central sections, rather than the reconstructed map or its associated Fourier coefficients, should play the role of data in model refinement.

The perspective that atomic structures should be refined against raw cryo-EM images was suggested in conclusion of an excellent recent review on cryo-EM refinement ^6^. We propose here directions for the explicit use of experimental images in cryo-EM refinement. We call G ^obs^_k_ the two-dimensional Fourier coefficients of the k ^th^ image and G ^calc^_k_ the corresponding calculated quantities, given by

$${\text{G}}^{\text{calc}}{\;}_{k}(\text{q})=\chi_{\text{k}}(|\text{q}|)\;\text{exp}(-2\pi i\text{q}{\text{o}}_{k})\;{\text{F(}R}_{\text{k}}\text{q),}\;(1)$$

where F is the three-dimensional Fourier transform of the model electrostatic potential, **R**_k_ the rotation that specifies the section orientation, **o**_k_ the origin shift that centers the section, χ _k_ the section’s associated CTF and **q** is a two-dimensional reciprocal vector. The mismatch between G ^obs^_k_ and G ^calc^_k_ may be used to define the experimental component of a refinement target function. The refinement target function should thus allow to couple the improvement of the model to that of the orientations, centers and CTF. Accordingly, the mismatch between G ^obs^_k_ and G ^calc^_k_ could be used to calculate an R-factor for structure assessment. Note that such criteria remain meaningful even in situations where maps are not of high quality, for example when the distribution of central sections is pronouncedly uneven.

By using experimental images as data, cryo-EM refinement procedures become thus quite similar to those used in X-ray crystallography, in spirit, in that the reconstructed maps are allowed to improve as model building and refinement proceed. A proof-of-principle assessment of the above proposal could be feasible based on available refinement software originally developed by X-ray crystallographers. Such software is mainly implemented in cartesian coordinates, but it can be anticipated that spherical coordinates will be more appropriate not only to handle rotations and interpolation of Fourier coefficients as required by Equation 1, but also to produce more accurate results ^7, 8^. Clearly, other not well understood aspects throughout the determination procedures will also need to be improved, such as dealing with errors on the detectors or multiple conformations of molecules ^6^, some of which may benefit from the proposed refinement target to yield statistically more robust structures. Ultimately, this may allow to better exploit the potential of the cryo-EM method and lead to a significant gain of accuracy of the high-resolution refinement protocol.

## Data availability

No data are associated with this article.
