# Supplementary material for: On the explicit use of experimental images in high resolution cryo-EM reﬁnement
Source: F1000Res. 2019 Nov 28;8:665. Originally published 2019 May 15. [Version 2] doi: 10.12688/f1000research.19230.2 (PMC6593323; doi:10.12688/f1000research.19230.2)
Supplement: Peer review history

# Peer review: Reviewer response for version 1

**DOI:** 10.5256/f1000research.21070.r50124

**Published:** 2019-06-25

**Authors:** S. Saif Hasan

**Affiliations:** 1 Department of Biochemistry and Molecular Biology, University of Maryland School of Medicine, Baltimore, USA

This article by Cherfils and Navaza provides an important path forward to model refinement in cryoEM, which deals with noisy data. The authors draw on rather rigorous methods of refinement and validation of coordinates in X-ray crystallography to propose the use of central section (or particles) in cryoEM as experimental data rather the reconstructed map.

 I would like to state that Michael Rossmann (Purdue University) would have been an appropriate reviewer for this paper as he was thinking on the same lines for the last few years. Unfortunately, Michael passed away recently. Nevertheless, this is a very interesting idea and provides a direction to incorporate true experimental data rather than depending on reconstructed maps, which could potentially have their own problems. 

 While I support indexing, I have a strong recommendation for the authors: the word "model" often does not translate well between crystallography and cryoEM. It could refer to coordinates in crystallography but in cryoEM, it could be the actual map or in later stages, the model of the atomic coordinates. Could the authors please ensure that they clarify this in the paper? On similar lines, "cryo-EM refinement" becomes ambiguous because it could refer to the reconstruction process (i.e., refinement of centers, orientation angles, defocus etc) or it could indicate the refinement of atomic coordinates against the map (or images as in this case). It would be extremely helpful to the readers if the authors could ensure that such confusion does not arise.

I confirm that I have read this submission and believe that I have an appropriate level of expertise to confirm that it is of an acceptable scientific standard.

# Peer review: Reviewer response for version 1

**DOI:** 10.5256/f1000research.21070.r48578

**Published:** 2019-05-17

**Authors:** Ignacio Fita

**Affiliations:** 1 Unit of Excellence María de Maeztu, Barcelona Molecular Biology Institut (IBMB-CSIC), Barcelona, Spain

The work by Cherfils and Navaza proposes to use the central sections from cryo-EM images, rather than the reconstructed maps or its associated Fourier coefficients, as the data for model refinement and validation. To achieve it, the section orientation and the origin shift that centers the section are incorporated in the evaluation of the two-dimensional Fourier coefficients of the model. In this way cryo-EM refinement can become similar to X-ray crystallography refinement.

 This is a very interesting proposal that could provide (implementation pending) an elegant approach to overcome major limitations in the accuracy and quality of the cryo-EM models obtained with the available methodologies.

 It might be good if authors could add some considerations about:

1. Noise in the individual cryo-EM sections could be high, which would degrade the accuracy of the observed two-dimensional Fourier coefficients. How these (large) errors could affect to the proposed refinement approach? 
2. With orientation and translation of individual sections applied to the model, how the “improved” experimental maps can be efficiently computed?

I confirm that I have read this submission and believe that I have an appropriate level of expertise to confirm that it is of an acceptable scientific standard.
